# Prenatal diagnosis of autosomal recessive osteopetrosis: a case report

**DOI:** 10.1186/1755-8166-7-S1-P125

**Published:** 2014-01-21

**Authors:** Mehul Mistri, Harsh Patel, Tanmay Tanna, Chitra Ankleshwaria, Frenny Sheth, Jayesh Sheth

**Affiliations:** 1 Institute of Human Genetics, FRIGE House, Ahmedabad-15, Gujarat, India; 2 National Institute of Technology Warangal, Andhra Pradesh 506004, India

## Background

Autosomal Recessive Osteopetrosis (ARO) or Malignant Infantile Osteopetrosis (MIOP) is a congenital disorder characterized by increased bone density on radiographs. It is known to be caused by mutations that cause loss of function in the *TCIRG1*, *CLCN7* or *OSTM1* genes. Classic symptoms include bone marrow failure, visual and hearing impairment, pancytopenia and extramedullary hematopoiesis leading to hepatosplenomegaly. The disease is lethal in infancy and the only curative treatment known is Hematopoietic Stem Cell Transplantation (HSCT). Prenatal diagnosis is done either by autoradiography or by identifying disease causing mutation.

## Case presentation

We present a family with consanguineous marriage with a male child born at full term with an uneventful incident and normal Apgar score. The child had normal milestones of development till two years of age. After this, he developed symptoms such as low platelet, low hemoglobin, splenomegaly and hydrocephalus. The patient was started with fortnightly blood transfusions. Clinically he was suspected to have osteopetrosis and investigated for mutations in TCIRG1 gene. Targeted exon sequencing identified the patient to be homozygous for a novel nonsense pathological mutation p.R426X (c.1276 C>T) in TCIRGI gene. He expired in the course of treatment at the age of 2.5 years. Subsequently, both parents were found to be heterozygous carriers for the same mutation. Prenatal counseling was advised for any future pregnancies. In the subsequent pregnancy, prenatal testing was done at 11 weeks of gestation using uncultured chorionic villus sample (CVS) to investigate for the aforementioned mutation. Analysis of genomic DNA obtained from uncultured CV has shown heterozygous status for *TCIRGI* p.R426X (c.1276 C>T) mutation. The family was informed of the carrier status of the fetus and their psychological trauma and stress was alleviated.

## Conclusions

Prenatal Diagnosis of Malignant Infantile Osteopetrosis (MIOP) through targeted sequencing of genomic DNA obtained from uncultured CV can be carried out once the mutation has been identified in an index case. This novel mutation also provides a new insight into the molecular basis of the disease which can be utilized for molecular diagnosis.

